# Myval Octacor and Octapro T*rans*-Catheter Heart Valves in Patients with Severe Pure Non Calcified Native Aortic Regurgitation

**DOI:** 10.1016/j.ijcha.2026.101924

**Published:** 2026-04-15

**Authors:** Alfonso Ielasi, Rodolfo Caminiti, Mariano Pellicano, Andrea Marrone, Dario Pellegrini, Carlo Banfi, Mattia Glauber, Maurizio Tespili

**Affiliations:** a U.O. Cardiologia Ospedaliera, IRCCS Ospedale Galeazzi Sant’Ambrogio, Milan, Italy; b Cardiology Unit, Department of Clinical and Experimental Medicine, University of Messina, Messina, Italy; c U.O. Cardiochirurgia Universitaria, IRCCS Ospedale Galeazzi Sant’Ambrogio, Milan, Italy; d U.O. Cardiochirurgia Mini-Invasiva, IRCCS Ospedale Galeazzi Sant’Ambrogio, Milan, Italy

**Keywords:** Transcatheter aortic valve implantation, Aortic regurgitation, Balloon-expandable *trans*-catheter heart valve

## Abstract

**Background:**

the treatment of high-risk or inoperable patients with severe, pure native non calcified aortic regurgitation (NCAR) poses unique procedural challenges for the *trans*-catheter technologies. In particular the lack of leaflet calcification and dilated aortic anatomies increase the risk of technical failure with the currently available *trans*-catheter heart valves (THV). This study sought to evaluate the feasibility, safety, and clinical outcomes following the off-label usage of the Myval Octacor and Octapro balloon-expandable (BE) THV in patients with pure NCAR.

**Methods:**

a retrospective cohort analysis was performed on consecutive high-risk surgical patients with severe, pure NCAR who underwent TAVI between January 2023 and June 2025 with the second (Octacor) and third (Octapro) generation of the Myval BE THV. Main end-points were technical and device success according to the Valve Academic Research Consortium (VARC) 3 criteria. The rates of permanent pace-maker implantation (PPI) and moderate-to-severe residual AR both, in-hospital and at the longest available follow-up were also evaluated.

**Results:**

during the study period, 32 patients (Octacor, n = 21 and Octapro, n = 11) with pure, NCAR were treated. Mean age was 79.3 ± 7.6 years while 81.2% were male. The mean left ventricular ejection fraction was 43.1 ± 7.4%. An XL size was implanted in 21 patients of whom 15 were 32 mm. The average percentage of THV oversize was 19.7 ± 6.9. Technical success was achieved in 87.5% of the cases due to partial migration of 4 THVs (all 32 mm implanted in large annuli) into the LVOT requiring rescue implantation of a second prosthesis. This strategy was effective in achieving a good result in 2 cases while in the remaining 2, the THVs embolized in the left ventricle requiring surgical conversion. No intraprocedural death or disabling strokes were reported. PPI rate at discharge was 9.4% while moderate residual AR was reported in 1 patient (including patient underwent successful rescue THV-in-THV). Device success was 87.5%. At 1-year follow-up, mortality and PPI rates were 3.1% and 15.6%, respectively with no severe residual AR cases reported.

**Conclusions:**

Octacor and Octapro BE THVs implantation in patients with pure NCAR is feasible and yields acceptable clinical outcomes in a high-risk population. Further studies are warranted to better understand the potential role of these THVs in this complex scenario.

## 1 Introduction

Transcatheter aortic valve implantation (TAVI) has become the standard of care for elderly patients with severe symptomatic aortic stenosis (AS) [1], [2], [3]. On the other hand, its use remains limited in patients with pure aortic regurgitation (AR) where guidelines recommend surgery as the gold-standard treatment [2]. This difference is mainly related to the anatomical AR features such as few or no calcium on the aortic valve leaflets, no fluoroscopic markers, and an enlarged aortic root/left ventricular outflow tract. As a result, the transcatheter heart valves (THV) may be prone to malpositioning, migration, embolization, and incomplete annular sealing, leading to significant residual AR. Because of this, a significant THV oversizing (20–30%) is necessary to secure the THV and reduce the risk of complication. Based on this, the required THV size is often beyond the range of most commonly available devices. Data on TAVI in pure non calcified AR (NCAR) are mostly deriving from the off-label use of various THVs [4], [5]. More recently, dedicated bioprostheses have shown improved outcomes compared to off-label devices [6]. However, these new THVs still have some limitations in terms of sizes and unavailability in everyday clinical practice. Since 2019 when received CE mark, the Myval balloon-expandable (BE) THV (Meril Life Sciences, Vapi, India) increased annular sizing up to 840 mm^2^ (diameter of 32.7 mm), addressing the anatomical demands of AR [6]. Although Myval THV was associated with interesting results in patients with severe NCAR, no data are available on the performance of its novel iterations (e.g. Octacor and Octapro) in this complex anatomical setting [7]. Aim of this study is to evaluate the feasibility, safety, and early clinical outcomes of TAVI following the use of Octacor and Octapro THVs in patients with pure, severe NCAR.

## 2 Methods

A retrospective cohort analysis was performed on consecutive high-risk surgical patients with pure, severe NCAR who underwent TAVI between January 2023 and June 2025 with the second (Octacor, from January 2023 to October 2024) and third (Octapro) generation of the Myval BE THV.

The study was conducted in accordance with the principles of the Declaration of Helsinki and Good Clinical Practice (GCP) guidelines.

### 2.1 Device

**Fig. 1:**
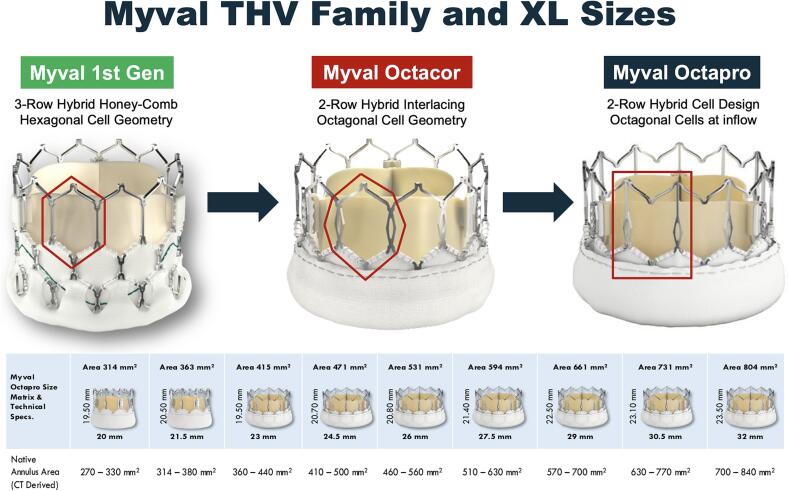
Myval transcatheter heart valve family.

Octacor and Octapro are the novel iterations of the Myval BE THVs characterized by a 2-row as compared to a 3-row cells design of the Myval generation 1. Octacor characteristics where already described elsewhere [8]. Octapro is the latest iteration of the family. It is composed by a cobalt alloy frame (total height from 19.5 to 23.5 mm) hosting an intra-annular bovine pericardium tri-leaflet valve. This novel THV is characterized by a 2 rows of interlacing hybrid cells design. The outflow zone (55% of the frame height) has V-shaped chevrons made by simple reversal of inverted V crowns while the inflow zone is composed by interlacing octagons. Based on this features Octapro foreshortens less (10–12%) during deployment as compared to Octacor (19–20%) and Myval generation 1 (21–24%). Likewise, Octacor and Myval, Octapro has an internal PET sealing skirt to minimize significant paravalvular leakage (PVL), while a higher (as compared to Myval gen 1) external skirt to plug micro-channels and further reduce PVL. Octapro is available in 9 sizes (from 20 mm to 32 mm) covering annular area up to 840 mm^2^ as well as Octacor and Myval. (Fig. 1) Octapro is advanced through the 14F expandable Python sheath (Meril Life Sciences) using the Navigator Pro delivery system (Meril Life Sciences) as its predecessors.

### 2.2 Study population

Patients were considered eligible for inclusion in this analysis in case of symptomatic, severe pure, native NCAR deemed unsuitable for surgical aortic valve replacement by the local Heart Team due to high or prohibitive surgical risk, as assessed by the Society of Thoracic Surgeons (STS) score and/or clinical judgment. Patients with severe AR and concomitant moderate or severe AS or significant calcific degeneration of the aortic valve leaflet were excluded. All patients provided written informed consent for the procedure and for the use of anonymized data for research purposes.

### 2.3 Pre-procedural assessment

Baseline evaluation included comprehensive clinical examination, laboratory testing, 12-lead electrocardiography, transthoracic echocardiography, and multi-slice computed tomography (MSCT) using a standardized TAVI acquisition protocol. The severity of NCAR was graded according to the current European Society of Cardiology guidelines [9], [10]. MSCT images were analyzed using dedicated software to measure annular, left ventricular outflow tract (LVOT), coronary ostia, and aortic dimensions, as well as calcium burden (Agatston score). New York Heart Association (NYHA) functional class was assessed at baseline.

### 2.4 Procedural details

All procedures were performed under local anesthesia plus conscious sedation and the support of the anesthesiologist in the lab was mandatory. All the patients needed to be suitable for *trans*-femoral TAVI with Octacor and Octapro. The interventional vascular access was planned to be obtained using percutaneous techniques as well as vascular closure was planned to be managed percutaneously using 2 proglides (Abbott Vascular). The choice of the ancillary access (femoral versus radial) was evaluated case by case as well as the protection of the interventional access leaving a 0.018″ wire. Unfractioned heparin was planned to be administered after proglide positioning in order to reach more than 250 s activated clotting time. THV sizing was based on MSCT annular and LVOT measurements, aiming for 20–30% oversizing [11]. A temporary pacing lead was planned to be positioned in the right ventricle from a femoral vein. Pacing rate during THV deployment was planned to be prolonged (15 to 30 s) at 200–220 beats per minute. The type (super-stiff versus extra-stiff) of the 0.035″ wire placed in the left ventricle was chosen according to the patients’ anatomy (tortuosity and/or horizontal aorta). Post-dilatation was left at the operator’s discretion according to the degree of residual AR or in case of suboptimal THV anchoring.

### 2.5 Study endpoints

All technical and procedural outcomes were adjudicated according to the Valve Academic Research Consortium-3 (VARC-3) criteria. Main endpoints were: VARC-3 technical success, defined as successful vascular access, delivery, and retrieval of the device; correct positioning of a single prosthetic valve into the proper anatomical location; and no procedural mortality; VARC-3 device success, defined as a composite of technical success and intended performance of the prosthetic heart valve (mean gradient < 20 mmHg, peak velocity < 3 m/s, Doppler velocity index ≥ 0.25, and less than moderate aortic regurgitation) at 30 days post-procedure. All-cause mortality, cardiovascular mortality, disabling stroke, myocardial infarction, new permanent pacemaker implantation (PPI), major bleeding, rehospitalization for heart failure, THV embolization or migration, need for second THV deployment, and conversion to surgery rates were also evaluated. All outcomes were reported in-hospital, at 30-day, and 1-year follow-up (the 1-year data representing cumulative event rates).

### 2.6 Follow-Up

Clinical follow-up was performed at 30-day and 1-year post-procedure through outpatient visits or telephone contact, including assessment of adverse clinical events and NYHA class.

### 2.7 Statistical analysis

Continuous variables were tested for normality using the Shapiro–Wilk test and are presented as mean ± standard deviation (SD) or median with interquartile range (IQR), as appropriate. Categorical variables are reported as counts and percentages. Given the observational design and limited sample size, analyses were primarily descriptive. When exploratory comparisons were performed, categorical variables were compared using Fisher’s exact test, and continuous variables using the Student’s *t*-test or Mann–Whitney *U* test, as appropriate. Statistical analyses were performed using SPSS Statistics version 29.0 (IBM Corp., Armonk, NY, USA).

## 3 Results

A total of 32 patients with severe, pure, native NCAR underwent TAVI using the Octacor (n = 21) or Octapro (n = 11) THV systems. The mean age was 79.3 ± 7.6 years while 81.2% of the subjects were male. The STS and EuroSCORE II scores were 4.5 ± 1.3 and 6.6 ± 3.2, respectively. The mean left ventricle ejection fraction (LVEF) was 43.1 ± 7.4% while 20% of the patients underwent prior cardiac surgery. Most of the patients were in advanced functional class at first clinical presentation: NYHA III 84.4% while NYHA IV 15.6% (Table 1).

**Table 1:** Baseline Clinical Characteristics.

Patients (n = 32)	
Age (years)	79.3 ± 7.6
Sex (male)	26 (81.2%)
BMI (kg/m2)	24.7 ± 3.7
Baseline serum creatinine (mg/dL)	1.4 ± 0.7
STS Score (%)	4.5 ± 1.3
EuroSCORE II	6.6 ± 3.2
Arterial hypertension	26 (81.2%)
Diabetes mellitus	7 (21.9%)
eGFR < 30 mL/min/1.73 m2	5 (15.6%)
Prior myocardial infarction	2 (6.2%)
Prior PCI	6 (18.8%)
Prior CABG	0 (0%)
Prior cardiac surgery	6 (18.8%)
Prior PPI	3 (9.4%)
Peripheral artery disease	0
Atrial fibrillation history	4 (16%)
Prior stroke	2 (9.5%)
Chronic obstructive pulmonary disease	1 (4%)
NYHA class	
*I*	0 (0%)
*II*	0 (0%)
*III*	27 (84.4%)
*IV*	5 (15.6%)
Left ventricle ejection fraction (%)	43.1 ± 7.4
Left ventricle end-diastolic diameter (mm)	60.8 ± 2.1
Mean aortic gradient (mmHg)	0.4 ± 1.1

BMI: body mass index; GFR: glomerular filtration rate; PCI: percutaneous coronary intervention; CABG: coronary artery bypass graft; PPI: permanent pacemaker implantation; NYHA: New York heart association.

Values are presented as mean ± standard deviation for continuous variables and as number (percentage) for categorical variables.

MSCT-assessed mean annular area was 587.9 ± 132.3 mm^2^ and annular perimeter 86.7 ± 9.9 mm. The average left main and right coronary artery heights were 14.7 ± 5.4 mm and 20.6 ± 6.3 mm, respectively. The mean ascending aorta diameter was 36.9 ± 5.2 mm and horizontal aorta was observed in 15.6% of patients. The “annulus/LVOT complex” shape was tapered in 25% of the cases. The mean Agatston calcium volume was 63.4 ± 150.4 mm^3^. All the patients had a tricuspid aortic valve (Table 2).

**Table 2:** Baseline computed tomographic and procedural characteristics.

Patients (n = 32)	
Annulus Area, mm2	587.9 ± 132.3
Annulus Perimeter, mm	86.7 ± 9.9
Left Main Height, mm	14.7 ± 5.4
Right Coronary Artery Height, mm	20.3 ± 6.3

Left Ventricle Outflow Tract shape	
*Tubular*	14 (43.8%)
*Flaired*	10 (31.2%)
*Tapered*	8 (25.0%)
STJ mean diameter, mm	34.7 ± 5.1
SoV mean diameter, mm	37.3 ± 4.9
Ascending aorta mean diameter, mm	36.9 ± 5.2
Max aortic diameter, mm	37.8 ± 4.9
Horizontal aorta (>47°)	5 (15.6%)
Bicuspid aortic valve	0 (0%)
Agatston Calcium Volume score	63.4 ± 150.4

THV size	
*30.5 mm*	6
*32 mm*	15
THV used Octacor/Octapro	21/11
% oversizing	19.7 ± 6.9
Extra-volume filling, mL	3.2 ± 3.0
Post-dilation	7 (21.9%)
Contrast volume used, mL	118.1 ± 62.4
Procedural time, minutes	67.1 ± 37.6
Fluoroscopy time, minutes	18.8 ± 8.6

STJ: sinotubular junction; SOV sinus of Valsalva; THV transcatheter heart valve.

Values are presented as mean ± standard deviation for continuous variables and as number (percentage) for categorical variables.

Safari wire (Boston Scientific) was used for THV deployment in 19 cases (59.4%) while Lunderquist (Cook Medical) in the remaining. A “buddy wire” strategy positioning a second Lunderquist protected by a pigtail in the ascending aorta was required in a patient with horizontal aorta and prior ascending aorta replacement with a prosthetic conduit. Python sheath was used in all the cases except one (where a 26F Dryseal −Gore Medical- was needed to advance a 32 mm Octacor in a patient with severe peripheral tortuosity). All the BE THVs were deployed directly (no pre-dilatation) under rapid (200–220 bpm) and prolonged (10-to-20 s) pacing. The majority (n = 21, 65.7%) of the THV implanted were extra-large (XL) and 15 over 21 were 32 mm size. In 10 cases an extra-volume (from 2 ml to 8 ml) was added to the deployment balloon in order to achieve a significant oversize (approximately 2.0–2.5% oversize increase per extra ml added). The mean oversize percentage was 19.7 ± 6.9. Post-dilatation was performed in 7 patients (21.9%) of whom 3 adding extra-volume (to the nominal) to the balloon which was the same used to deploy the THV plus a maximum of 10 ml.

**Fig. 2:**
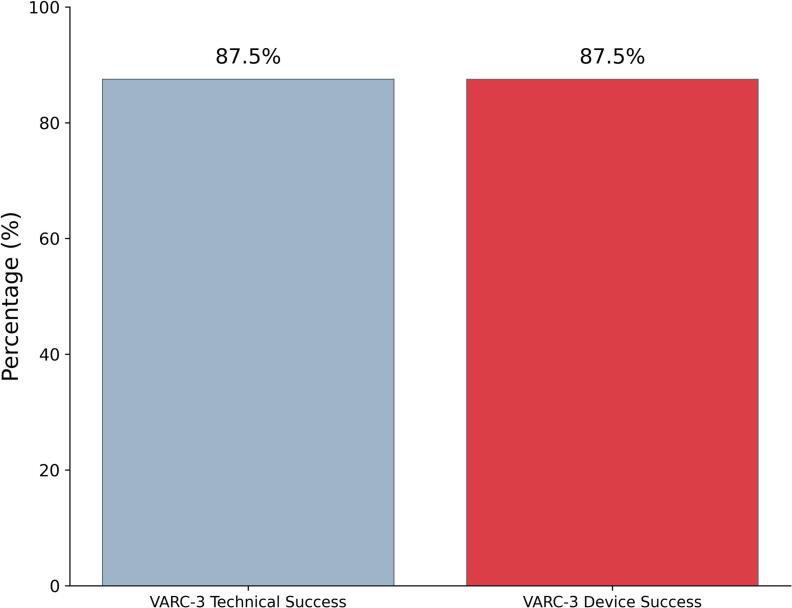
VARC-3 technical and device success rates.

VARC-3 technical success was achieved in 87.5% of the cases (Fig. 2). Partial THV migration in the LVOT after deployment occurred in 4 patients (16%) requiring a 32 mm Octacor. In all these cases post-dilatation with extra-volume into the balloon was performed in order to overexpand and anchor the THV. As the THVs were still not stable in position after post-dilatation, a second 32 mm THV was implanted (with extra-volume in the balloon) slightly higher into the first THV with the aim to achieve a more stable anchoring. In 2 cases this strategy was effective in achieving a good final result while in the remaining 2, THVs embolized into the left ventricle following a THV-in-THV post-dilatation. In both cases conversion to surgery was needed. One of these patients survived the event while the other one died after 21 days because of sepsis (in-hospital death) (Table 3).

**Table 3:** Anatomical and procedural characteristics of the patients with left ventricular THV embolization.

	Annular Area (mm^2^)	Annular/LVOT Complex Shape	THV Type/Size	THV Oversize Nominal Volume (%)	Extra-Volume (ml)	Post-Dilatation Balloon Overfilling	Second THV Implanted Size	Second THV Overfilling	Outcome
Pt 1	663	tubular	Octacor 32 mm	21	2	Yes	Octacor 32 mm	Yes (+ 2 ml)	Surgical Conversion/Death
Pt 2	667	tapered	Octacor 32 mm	21	0	Yes	Octacor 32 mm	No	Surgical Conversion/Survived
Pt 3	787	tapered	Octacor 32 mm	2	8	Yes	Octacor 32 mm	Yes (+10 ml)	Survived No PVL
Pt 4	614	tubular	Octacor 32 mm	26	0	Yes	Octacor 32 mm	Yes (+2 ml)	Survived No PVL

LVOT: left ventricle outflow tract; THV: transcatheter heart valve; PVL: paravalvular leak.

**Fig. 3:**
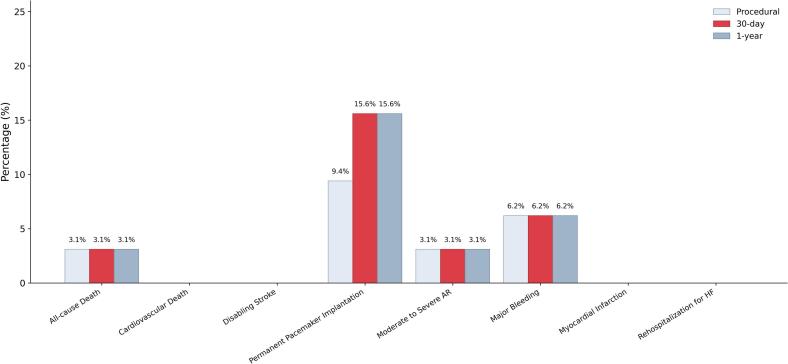
Clinical in-hospital and follow-up outcomes. HF Heart Failure, PPI Permanent Pacemaker Implantation. *Major Bleeding according to VARC-3 criterion.

New PPI was required in 9.4% of patients at discharge, moderate residual AR at discharge was reported in 1 patient only (including the patients underwent successful acute THV-in-THV) while no severe residual AR. Any major vascular complication was reported as well as disabling stroke nor cardiovascular death (Fig. 3 and Table 4).

**Table 4:** Procedural outcomes and in-hospital events.

VARC-3 technical success	28 (87.5%)
Procedural death	0 (0%)
All-cause death	1 (3.1%)
Cardiovascular death	0 (0%)
Disabling stroke	0 (0%)
Myocardial infarction	0 (0%)
New permanent pacemaker implantation	3 (9.4%)
Major bleeding	2 (6.2%)
Rehospitalization for HF	0 (0%)
Left Ventricle THV embolization *	1 (3.1%)
Need for second THV deployment	4 (12.5%)
Conversion to surgery due double THV embolization	2 (6.2%)
Moderate to severe aortic regurgitation	1 (3.1%)

VARC: Valve Academic Research Consortium; THV: transcatheter heart valve; HF: heart failure.

*Valve embolization/migration defined per VARC-3.

At 30-day follow-up, VARC-3 device success was 87.5% (Fig. 1). All-cause mortality remained 3.1%, with no cardiovascular deaths, disabling strokes, rehospitalizations for heart failure while 1 additional PPI was required. No cases of more than moderate residual AR were reported (Fig. 3).

At 1-year follow-up, outcomes remained stable. All-cause mortality was 3.1%, with no additional cardiovascular deaths, disabling stroke or heart failure hospitalizations. The cumulative PPI rate remained also stable (15.6%) as well as moderate or severe residual AR. All 1-year outcomes are reported as cumulative events occurring from the index procedure through follow-up, including in-hospital events (Fig. 3 and Table 5).

**Table 5:** Outcomes at Follow-up.

	*30-day*	*1-year*
VARC-3 Device Success	28 (87.5%)	—
All-Cause Death	1 (3.1%)	1 (3.1%)
Cardiovascular Death	0 (0%)	0 (0%)
Disabling Stroke	0 (0%)	0 (0%)
Myocardial Infarction	0 (0%)	0 (0%)
Permanent pacemaker implantation	5 (15.6%)	5 (15.6%)
Major Bleeding	2 (6.2%)	2 (6.2%)
Rehospitalization for HF	0 (0%)	0 (0%)
Moderate to severe aortic regurgitation	1 (3.1%)	1 (3.1%)

HF: heart failure; VARC: Valve Academic Research Consortium.

Outcomes are reported as cumulative events occurring from the index procedure through 1-year follow-up, including in-hospital events.

## 4 Discussion

Our experience with the use of the Myval Octacor and Octapro BE THV for the treatment of severe, pure native NCAR demonstrated acceptable technical and device success rates with no cases of severe residual AR up to 1-year follow-up.

TAVI was designed and validated as a treatment for severe AS. Based on the different anatomical scenario, this approach cannot be immediately applied to NCAR. AS is often characterized by extensive calcifications at both the annulus and leaflet levels, which facilitate the anchoring of both self-expanding (SE) and BE THVs, as well as provide a fluoroscopic landmark for guiding the implant. Patients with pure NCAR often exhibit little to no calcium, elliptical and large annulus, dilation of the aortic root and ascending aorta, and a large stroke volume with turbulent regurgitant jet. These characteristics represent a challenge in the transcatheter treatment of this valvular heart disease. The main issue when dealing with pure NCAR is anchoring the THV to the annulus. Due to the lack of calcium, a significant THV oversizing (more than 20%) is necessary to secure the THV and reduce the risk of migration, embolization, and/or significant residual AR [12], [13]. As a result, the required THV size is often beyond the range of commonly available devices representing the most common anatomical contraindication for TAVI, particularly in the AR setting. The need for substantial oversizing is also theoretically associated with a higher risk of advanced conduction disturbances, potentially necessitating PPI [14].

The feasibility of TAVI in NCAR using non-dedicated THVs has been demonstrated in multiple registries and case series [15].

Early experiences with the BE SAPIEN (Edwards Lifesciences) and the SE CoreValve/Evolut (Medtronic) platforms confirmed procedural viability but were associated with a high incidence of second THV implantation, device migration, and moderate-to-severe residual AR [4], [16]. Subsequent studies by De Backer et al. and Yoon et al. showed that newer-generation THVs significantly improved device success rates and reduced complications compared to earlier platforms, although important procedural risks persisted [4], [15].

Nonetheless, it remains unclear which THV system is best suited for NCAR. While SE THVs such as Evolut offer advantages including retrievability and larger size availability, they have been associated with lower device success rates, increased need for second THV implantation, greater postprocedural AR, and higher PPI rates [15]. These data suggest that BE THVs may offer more stable anchoring due to higher radial force and expansion, albeit at the cost of being non-repositionable and dependent on annular oversizing.

More recently, the FRANCE TAVI (BE THV 45%) and PANTHEON (BE THV 34%) studies provided further insights into the pure AR population. Both reported technical success rates of 85.5% and 83.6%, respectively, with THV embolization and need for a second prosthesis implantation as major procedural challenges. FRANCE TAVI, which included an older population (81 vs. 79 years), observed a higher PPI rate (36% vs. 22%) and worse long-term mortality (53.5% at 4 years), while PANTHEON emphasized the prognostic impact of THV embolization and identified post-dilatation as a key predictor of device migration [17], [18].

Myval BE THV is the only prosthesis available in extra-large sizes (30.5 mm and 32 mm while also 35 mm available in India) allowing the treatment of larger annuli as compared to the other commercially available THVs. This feature poses a unique advantage of this platform for treating pure NCAR where significant oversizing is needed to anchor the THV on the aortic wall. Technical success following the use of Myval generation 1 BE THV in patients with pure NCAR and large annuli (mean area 638.5 mm^2^ versus 510 mm^2^ in the PANTHEON) was 94.7%. A tapered anatomy (left ventricle outflow tract −LVOT- larger than the annulus) was detected as an unfavorable scenario for THV anchoring being associated with a higher risk of embolization compared to the tubular and flared anatomies [7].

**Fig. 4:**
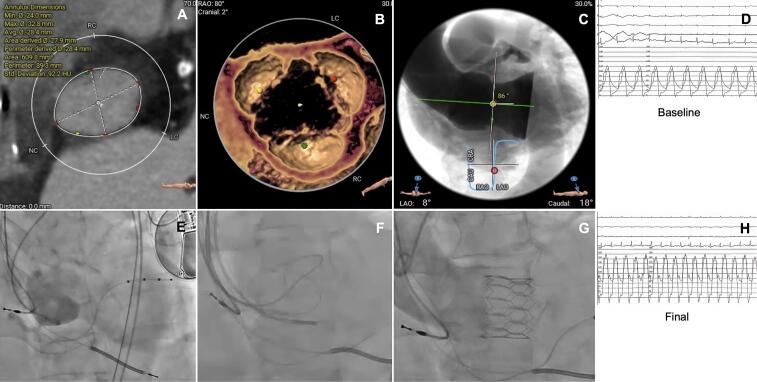
TAVI using Octapro in a patient with pure, severe non calcified aortic regurgitation Legend: A) MSCT assessed large aortic annulus; B) Non calcified trileaflets aortic valve; C) Extremely horizontal aorta; D) Hemodynamic at baseline; E) Baseline aortogram; F) Fluoroscopic markers to identify the coplanar view; G) Octapro 30.5 mm (20.9% oversize versus annular area) in position; H) Final hemodynamics.

Our study is the first specifically evaluating the performance of the latest iterations of the Myval BE THV family in the challenging anatomical scenario of NCAR. Main difference of Octacor and Octapro versus Myval is the 2-rows rather than the 3-rows frame design which is associated with less THV foreshortening.(Fig. 1, Fig. 2, Fig. 3, Fig. 4) Also, the outer skirt is slightly larger in the novel iterations theoretically increasing the sealing properties of the THV [8].

In our cohort (mean annular area 587.9 mm^2^), THV migration/malpositioning occurred in 4 (12.5%) cases (annular areas ranging from 614 mm^2^ to 787 mm^2^), all requiring post-dilatation and second 32 mm THV-in-THV implantation. Among these patients, 2 (6.2%) experienced THV-in-THV embolization in the left ventricle requiring surgical conversion while 2 survived the event without residual severe AR.

Unstable anchoring is the Achilles Heel of each conventional THV used off-label in this setting. In particular, the treatment of large or XL annuli (most of them untreatable even with dedicated THVs) may be associated with a higher risk of THV embolization. By analyzing the cases of Myval migration (Table 3) we can highlight that the THV oversize is exceeding 25% in one case only (tubular anatomy). This is probably a crucial aspect to be considered upfront particularly in patients with XL and tapered anatomies which should not be considered good candidates for the (off-label) procedure in case that minimum oversize (25%) cannot be reached out. In our view, the need for post-dilatation and second THV implantation are the consequences of an incorrect patient selection rather than wrong procedural techniques. Anyway, in patients properly selected for the procedure, we suggest to perform post-dilatation (oversized balloon) and eventual THV-in-THV implantation (again oversized balloon) in case of significant residual AR and/or unstable first THV anchoring if its position is not lower than 50:50 (aorta:left ventricle).

While annular oversizing by 25–30% may reduce the likelihood of embolization, it simultaneously raises concerns regarding annular rupture, aortic dissection, and particularly conduction disturbances [11], [12], [19]. As in the Myval generation 1 AR study, also in our study no cases of periprocedural aortic rupture/dissection were reported as well as no severe residual AR at discharge, 30-day and 1-year demonstrating a high safety profile and sealing properties of both BE THVs in this setting (almost one fourth of our population had horizontal aorta and tapered LVOT anatomy).

On the other hand, PPI rates were relatively high: 9.4% at discharge and 15.6% at 30-day and 1-year versus 13.4% at discharge and 22.2% at 1-year in the Myval gen-1 study [7].

Dedicated THVs for AR, such as the CE marked JenaValve Trilogy (JVT) and the J-Valve (only available in China and in the U.S. for investigational purposes), may overcome some of the limitations associated with off-label use of conventional THVs [20], [21]. In the PURPOSE study, JVT (n = 88) was compared with off-label THVs (BE n = 70; SE n = 98) in 256 inoperable AR patients. JVT demonstrated superior VARC-3 technical success and device success, along with significantly lower rates of THV embolization and moderate or greater residual AR. However, 1-year clinical outcomes, including all-cause mortality and heart failure rehospitalization, were similar between groups [22].

A recent *meta*-analysis by Samimi et al. involving 2,162 high-risk AR patients showed that dedicated THVs were associated with lower 30-day and 1-year mortality, higher device success, and fewer complications, including residual AR, THV embolization, and PPI compared to off-label THVs [6]. However, sizes of dedicated THVs are still limited (JVT is available in 3 sizes only matching perimeters from 66 to 90 mm) as well as their availability outside U.S. and China.

**Fig. 5:**
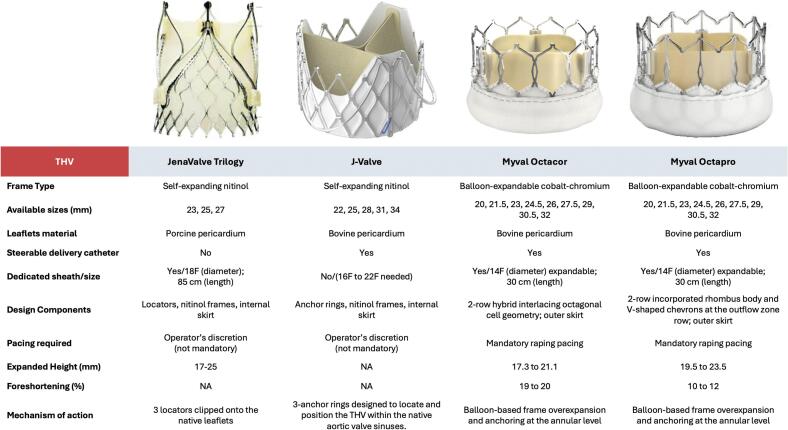
Main differences among dedicated *trans*-catheter heart valves versus Octapro and Octacor.

On the other hand, the Myval THV family, which is available in all continents, provides XL sizes (matching annular areas up to 840 mm^2^ at nominal volume and even more adding extra milliliters of solution to the balloon) allowing to treat a broader proportion of inoperable/high-risk AR patients. In consideration of all these aspects, dedicated THVs cannot be considered today as a realistic option for patients needing this therapy while the “conventional” Myval THV family, will continue to answer the unmet clinical need of AR treatment on a large scale. Fig. 5.

In this view, our study adds further insight into the feasibility of TAVI for NCAR using novel generations BE THV, yet also reinforce the need for device improvements or the development of more dedicated solutions. Until then, careful case selection (excluding tapered LVOTs and/or anatomies where a proper oversizing −25–30%- cannot be achieved) and planning remain essential when using non-repositionable systems like Octacor/Octapro in this challenging anatomy. While Myval XXL sizes (e.g. 33.5 and 35 mm) will help treating even larger anatomies in AR patients [23], [24], further studies with larger patient cohorts and extended follow-up are warranted to more comprehensively assess the performance and durability of these novel generation BE platforms.

## 5 Limitations

This study has several limitations that warrant consideration. First, it was a retrospective analysis which may limits the generalizability of the findings even if all the NCAR cases underwent TAVI during the study period were treated with Octacor and Octapro. Second, the sample size is small, reflecting the rarity of TAVI in pure NCAR, and precluding robust statistical comparisons or subgroup analyses. Third, clinical and echocardiographic follow-up was limited to 1 year, and longer-term durability and outcomes of the novel Myval BE THV family iterations in this remain unknown. Finally, the findings should be interpreted cautiously given the off-label use of the THVs in this particular complex subset of patients.

## 6 Conclusions

TAVI using Myval Octacor and Octapro in patients with severe, pure native NCAR is associated with acceptable technical and device success with no severe residual AR. While longer-term data and direct comparisons with dedicated THV technologies are awaited, the novel iterations of the Myval BE THV family represent a valuable and available alternative in this complex anatomical setting.

Declarations

## CRediT authorship contribution statement

**Alfonso Ielasi:** Writing – original draft. **Rodolfo Caminiti:** Writing – review & editing. **Mariano Pellicano:** Supervision. **Andrea Marrone:** Visualization. **Dario Pellegrini:** Validation. **Carlo Banfi:** Visualization. **Mattia Glauber:** Supervision. **Maurizio Tespili:** Methodology.

## Funding

This research did not receive any specific grant from funding agencies in the public, commercial, or not-for-profit sectors.

The authors take responsibility for all aspects of the reliability and freedom from bias of the data presented and their discussed interpretation

## Declaration of competing interest

The authors declare that they have no known competing financial interests or personal relationships that could have appeared to influence the work reported in this paper.
